# Ambient temperature and non-communicable disease presentations and mortality in a Pakistani ED

**DOI:** 10.1016/j.joclim.2026.100768

**Published:** 2026-10-28

**Authors:** Syed Ghazanfar Saleem, Zainab Tariq Soomro, Saima Ali, Ammad Fahim, Megan Rybarczyk, Joseph Dominick Ciano

**Affiliations:** a Indus Hospital and Health Network, Department of Emergency Medicine, Karachi, Pakistan; b Indus Hospital and Health Network, Office for Innovation, Research and Commercialization, Karachi, Pakistan; c Hospital of The University of Pennsylvania, Department of Emergency Medicine, Philadelphia, PA, USA

**Keywords:** Environmental health, Emergency medicine, Climate change, Public Health

## Abstract

**Introduction:**

Non-communicable diseases (NCDs) contribute significantly to the global health burden, and their outcomes may be influenced by temperature variations. This study evaluated the impact of ambient temperature on emergency department (ED) visits and associated mortality of certain NCDs in a low-resource tertiary care setting in Pakistan.

**Methods:**

An electronic health records (EHR) review was conducted for adults (≥18 years) presenting to Indus Hospital and Health Network (IHHN) ED (Karachi, Pakistan) from 2014 to 2024. Patients with hypertension (HTN), diabetes mellitus (DM), ischemic heart disease (IHD), obstructive lung disease (OLD), chronic kidney disease (CKD), or cerebrovascular accident (CVA) were included. Daily ambient temperatures were sourced from OpenWeather. Visit and mortality rates were stratified across temperature categories. Temperature effects on mortality risk were modeled using the Accelerated Failure Time (AFT) model. Autoregressive Distributed Lag (ARDL) models evaluated the delayed effect of extreme heat (≥31 °C) on daily mortality rates.

**Results:**

Of the 1003,894 adult ED patients, NCD visits varied from 6–60/day at various temperatures with visits decreasing with rising ambient temperatures. Mortality rates rose at temperature extremes (<20 °C and ≥31 °C). Time to death or discharge decreased by 14–25% in higher temperatures (≥31 °C) when compared with temperatures between 25.5–29.5 °C for HTN, DM, IHD, and CVA, as confirmed through the AFT model. ARDL models confirmed a delayed effect of extreme temperature (≥31 °C) on mortality rates for HTN, IHD, and CVA.

**Conclusion:**

Temperature extremes were found to significantly influence NCD-related ED burden and outcome. The findings from the study highlight the need for climate-adaptive emergency care strategies in low-resource settings.

## 1 Introduction

The role of seasonal variability in the prevalence of infectious diseases is well known, but the relationship between ambient temperature and non-communicable diseases (NCDs) is less understood. Multiple studies have demonstrated a correlation between climate and infectious diseases [[1], [2], [3], [4]]. Malaria and other vector-borne illnesses are common during humid, warmer seasons, while influenza, rotavirus, and pneumococcal infections are encountered more during colder weather [3,4]. Studies from around the globe have shown increased incidence, exacerbations, hospitalizations, and poor outcomes related to cardiovascular diseases (CVD) and cerebrovascular accidents (CVA) during cold months [[5], [6], [7], [8], [9]]. Some theories behind this trend include cold-related sedentary lifestyles, dietary changes, differences in catecholamine and vasopressin release, and increased systolic blood pressure in colder temperature [7,10,11].

Additional studies have demonstrated poor outcomes and an increased risk of hospitalization for ischemic heart disease (IHD) and CVA during heat waves [[12], [13], [14]]. This is especially true for populations with less capacity to adapt to extremes in temperature, like pediatric and geriatric populations [11,13]. Other NCDs, like obstructive lung disease (OLD), diabetes mellitus (DM), and acute kidney injury (AKI), have shown increased exacerbations, and disease-related hospitalizations during cold months [8,9,[15], [16], [17], [18], [19], [20], [21]]. Seasonal variations in NCD prevalence, mortality, and admission rates are reported globally, but similar data from Pakistan is limited [[5], [6], [7], [8], [9],[15], [16], [17], [18], [19], [20], [21]].

The relationship between temperature and disease burden is especially relevant to countries, like Pakistan, that are disproportionately affected by climate change and extreme weather events [12,22,23]. A retrospective review from Khyber Pakhtunkhwa province in Pakistan highlighted high mortality due to CVD, respiratory, and kidney diseases in winter months with a peak in all-cause mortality [16]. Other studies in Pakistan have shown a seasonality to NCDs with increased admissions for DM and IHD in colder months [14,17]. ED specific studies in China, Bangladesh, and the United States have demonstrated correlations between extremes in temperature and increased ED visits due to CVD, IHD, cerebrovascular disease, heart failure, respiratory conditions, and renal dysfunction [[24], [25], [26]]. However, comprehensive ED-based data investigating the role of ambient temperature on NCDs in Pakistan are limited.

The aim of this study was to investigate the influence of ambient temperature on certain NCDs presenting to the ED at Indus Hospital and Health Network (IHHN) in Karachi, Pakistan and to observe its impact on NCD-related mortality.

## 2 Methods

### 2.1 Study design and setting

The study utilized a retrospective cross-sectional design at IHHN, Karachi, Pakistan. IHHN Karachi serves as the head office of the philanthropic network and caters to people from across the city. Due to the free-service model, the ED has an average daily volume of >500 patients, the majority of whom belong to marginalized communities and have negative social determinants of health. Medical care is delivered based solely on financial donations, and no government subsidies are used to provide care. The hospital underwent expansion during the study, with an increase in the number of ED and in-patient beds and services offered. The coastal megacity of Karachi faces distinct meteorological challenges, experiencing oppressive maritime humidity, severe urban heat island effects, and a growing threat from unpredictable Arabian Sea cyclones [27].

### 2.2 Ethics statement

The study was deemed exempt from full IRB review at both IHHN (IRB# 2024_11_025) and the University of Pennsylvania (IRB protocol # 857689) as the criteria for human subject research were not met. Patient records were de-identified by the electronic medical record team before the dataset was made available to the study authors. The authors did not have access to identifying information (patient name, phone numbers, address, or other identifying information) during or after data collection/extraction.

### 2.3 Participants

Charts of patients ≥18 years of age presenting to the IHHN ED were queried using the electronic health record from July 1, 2014 - June 30, 2024. Documentation of HTN, DM, IHD, OLD, CKD, and CVA in the history of present illness (HOPI) or primary ED diagnosis was included. Charts not listing these NCDs were excluded due to limited in-patient bed capacity and non-availability of certain specialties at IHHN throughout the study period.

NCDs were identified using a natural language processing (NLP) approach that employs predefined patterns (implemented with regular expression (regex)) to extract information from text. The rules were designed to capture common variations in medical terminologies, abbreviations, negative expressions, and common spelling errors. If a patient was marked as having a certain NCD, then for all subsequent visits, it was assumed that the patient still had that NCD. To assess the performance of this tagging approach, the regex-extracted annotations were compared against a manually annotated gold standard subset of 2,500 records created by two ED physicians. The annotations achieved ≥ 0.90 on accuracy, precision, recall, and F1 score for all NCDs when compared against the reviewers’ records.

### 2.4 Variables and data sources

Patients’ biological sex, age, date of ED visit, hospital length of stay (LOS), triage acuity (Manchester triage criteria with P1 as the highest acuity and P5 as the lowest acuity) [28], HOPI, ED primary diagnosis, all-cause mortality in ED, and all-cause mortality associated with ED-inpatient admission were extracted from the electronic health record. Missing data were removed if either triage acuity, LOS or discharge status were not recorded.

Hourly ambient temperatures in Karachi, Pakistan during the study period were obtained from OpenWeather, an online software that provides historical weather data based on satellites, radars, and a large network of weather stations globally [29,30]. The ED in the study primarily served Karachi; therefore, temperature exposure was considered to be uniform across the study population.

A 99.9 percentile threshold was used as the upper limit for age and LOS. For descriptive analysis, age and LOS were converted from numerical to categorical variables. Age was categorized into ten-year intervals, while LOS was categorized in <4, 4–7, 8–11, 12–23, >24 h. Ambient temperature was aggregated from mean hourly to mean daily temperature. Temperature groups (<20, 20-<25.5, 25.5-<29.5, 29.5-<31, ≥31 °C) were formed based on the past ten-year percentiles (10th, 33rd, 66th, 90th) to enhance understanding of their differences. To account for a full calendar year, years were grouped from July to June (e.g. 2014–15 included visits from July 1, 2014, through June 30, 2015).

### 2.5 Data analysis

Python software version 3.12.3 was used for analysis. NCDs were categorized into diagnostic groups. A patient with two or more NCDs listed in the ED charts was included in each NCD subgroup. For example, a patient with HTN and DM was counted in both HTN and DM subgroups. Each NCD group was compared to the ambient temperature data. U-shaped curves were used to investigate possible trends.

Variations in the prevalence of NCDs over the study period were considered during the analysis to limit confounding, so visit and mortality rates were recorded as percentages, rather than counts. Visit rates were calculated as the number of visits with an NCD divided by total ED visits. Mortality rate was calculated as the number of expired patients with an NCD divided by the total ED visits for the NCD. 95% confidence intervals for these rates were calculated using Wilson confidence intervals.

To understand the relationship between ambient temperature and patient survival, an Accelerated Failure Time (AFT) model was run. In this model, the time variable was LOS and event was in-hospital death. To determine the most appropriate parametric form for the survival distribution, different AFT models (Weibull, exponential, log-normal, and log-logistic) were fitted, and the model with the lowest Akaike Information Criterion (AIC) was selected. The final model was built on Weibull distribution with separate models for each NCD. Robust standard errors were used to account for multiple visits by the same patient. All models included ambient temperature groups at presentation, age in years, sex, and triage acuity as covariates. Effect estimates were reported as time ratios with 95% confidence intervals. If the time ratio for a covariate is 0.5, it means that the acceleration to the event, which in this study’s scenario is death, will happen 50% faster than the reference groups. Conversely, a time ratio of 1.5 means it would be 50% slower.

To evaluate the short-term lagged association between extreme temperature (≥31 °C) and mortality rate for each NCD, the Autoregressive Distributed Lag model was used. The model’s independent variables included lag terms from L0 (same day) to L7 (7-day prior to the ED visit) for exposure while the dependent variable was daily mortality rate. Effect estimates were reported as beta coefficients with a 95% confidence interval.

## 3 Results

### 3.1 Demographic details

Out of the 1.73 million visits of patients who presented to the IHHN ED during the ten-year study period, 1.04 million patients were ≥ 18 years of age. After removing records with missing information, 1,003,894 patient visits remained for analysis (Appendix 1). There were 516,180 (51.4%) females in the study cohort. HTN was the most prevalent subgroup (20.07%), followed by DM (14.24%), IHD (8.70%), OLD (4.18%), CKD (2.71%), and CVA (2.46%). The remaining 47% of visits did not have the NCDs under study. Images displaying the distribution of patient visits for NCDs and repeated visits by patients are included as supplementary data in Appendices 2 and 3, respectively.

### 3.2 Trend of all patient visits at IHHN ED

Total annual ED patient visits increased over the decade from 80,896 visits/year in 2014–15 to 103,208 visits/year in 2023–24. Two surges in ED visits in 2017 and 2021 occurred during an ED infrastructure expansion, and one dip in 2020 occurred during COVID-19 quarantine restrictions. Over the years, average daily visits increased with a rise in the mean daily temperature. 37.28% of visits fell between temperatures 25.5–29.5 °C.

### 3.3 Trend of patient visits at IHHN for NCDs

For all NCDs in this study, an increasing trend in patient visits was noted (Fig. 1, Table 1).

**Fig. 1:**
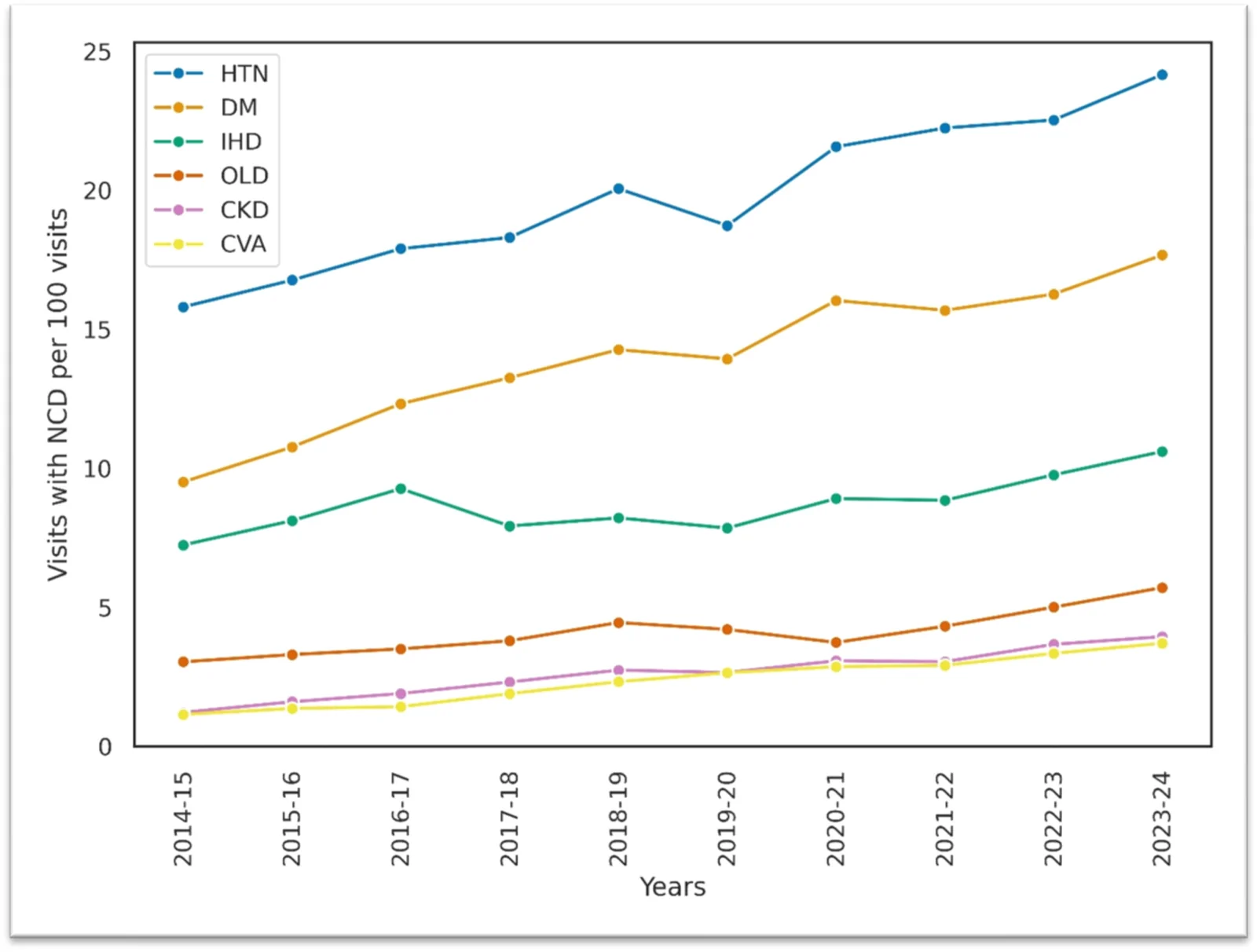
Pattern of non-communicable disease visits at IHHN ED between 2014–2024. HTN: Hypertension, DM: Diabetes Mellitus, IHD: Ischemic Heart Disease, OLD: Obstructive Lung Disease, CKD: Chronic Kidney Disease, CVA: Cerebrovascular Accident.

**Table 1:** Stratification of non-communicable disease patients by daily mean ambient temperature.

Mean daily ambient temperature ( °C)		<20	20–25.5	25.5–29.5	29.5–31	≥31
Total visits		92,654	222,434	374,281	199,782	114,743
Number of days at listed temperature		369	830	1339	698	414
Gender (F)		47,803	115,775	190,836	102,842	58,924
Mean age in years (SD)		41.63(17.00)	41.16(16.77)	40.14(16.35)	39.91(16.23)	40.09(16.36)
Mean visits/day (SD)	All	251.1 (56.64)	267.99 (58.63)	279.52 (59.33)	286.22 (62.21)	277.16 (67.46)
	HTN	57.03 (16.92)	58.52 (18.21)	54.55 (17.29)	53.42 (18.79)	51.98 (19.68)
	DM	39.38 (13.08)	40.37 (14.45)	38.75 (14.14)	38.52 (14.66)	38.91 (15.13)
	IHD	25.1 (7.83)	25.76 (7.84)	23.62 (7.45)	22.8 (7.59)	22.08 (8.17)
	OLD	13.06 (5.73)	13.5 (5.95)	10.99 (5.1)	10.39 (5.27)	9.67 (5.02)
	CKD	8.43 (4.25)	7.61 (4.36)	7.18 (4.19)	7.31 (4.21)	7.38 (4.29)
	CVA	7.35 (4.11)	7.11 (4.22)	6.47 (3.99)	6.44 (3.98)	7.05 (4.36)

HTN: Hypertension, DM: Diabetes Mellitus, IHD: Ischemic Heart Disease, OLD: Obstructive Lung Disease, CKD: Chronic Kidney Disease, CVA: Cerebrovascular Accident, SD: Standard Deviation.

ED visits, sex, LOS, and triage acuity for each NCD stratified by temperature are described below and are detailed in Appendix 4.

### 3.4 Hypertension (HTN)

Most HTN patient visits were female (60%) and tended to be older with patients >60-year-old comprising the highest number of visits from this group (38–42%). Most visits had LOS of <4 h (43–48%) with only 4.5% of the patients’ LOS >24 h. This included those who were admitted to inpatient services. The highest (48–52%) number of patients were triaged as P3, followed by P2, P1, and P4/P5. Triage acuity levels P1 and P4/5 followed a U-shaped curve while P2 and P3 followed an inverted U-shaped curve with respect to temperature. The percentage of expired patients (<3%) followed a U-shaped curve with around a 1% increase from the middle temperature group (25.5–29.5 °C) to the highest temperature group (≥31 °C).

#### 3.4.1 Diabetes mellitus (DM)

Across all temperatures, female patients with DM presented more often (55%-57%) than male patients with DM (43–45%). The oldest age group (≥60 years) remained prevalent among all temperatures; however there was a downward trend in presentation (from 41.5% to 38.2%) as temperature increased from <20 °C to ≥31 °C. Most patients were discharged within four hours across all temperature groups (42–47%) and discharge after 24 h remained low (4–6%). Triage acuity followed the same pattern throughout the temperature groups with P3 (48–52%), followed by P2, P1 and P4/5. There was a U-shaped trend in P1 triages (<20 °C: 11.1%, 25.5 °C -29.5 °C:8.5%, ≥31 °C: 10.6%). The percentage of expired patients followed a U-shaped curve with around a 0.7% increase from 25.5–29.5 °C to ≥31 °C.

#### 3.4.2 Ischemic heart disease (IHD)

Males (∼57%) comprised most IHD visits across all temperature groups. Most patients were over 60 years of age (45–50%), and <2% of the visits were between 18 and 29 years old. Most visits (41–45%) lasted less than four hours across temperatures. As the temperature rose, there was a 3% decrease in the LOS <4 h and a slight increase (1.1%) in LOS >24 h. At all temperature groups, the most prevalent triage acuity was P2 (43–47%), followed by P3, P1, and P4/5. P1 had the highest percent (11.4%) at the coldest temperature (<20 °C) compared to other temperatures, following a skewed U-shaped curve (towards <20 °C). All-cause mortality rates were between 2.3% and 3.1% across all temperatures and followed a U-shaped curve.

#### 3.4.3 Obstructive lung disease (OLD)

Males and females were equally distributed across all the temperature groups with the majority of patients >60 years (34–38%). OLD was the most prevalent NCD in the 18–29 age group with most visits (14.7%) between 25.5–29.5 °C. Around half (45%) of visits were discharged within four hours while there was a 1.9% increase in visits lasting >24 h from <20 °C (4.3%) to ≥31 °C (5.4%). Most of the patients were triaged as P2 (39–43%) with P2 and P3 being almost equal at ≥31 °C. P1 presentations went from 22.5% at <20 °C to 17.7% at ≥31 °C. There was a U-shaped trend in all-cause mortality, with 2.2% patients expiring at <20 °C versus 1.5% expiring at 25.5–29.5 °C and 1.9% at ≥31 °C.

#### 3.4.4 Chronic kidney disease (CKD)

CKD visits were distributed evenly across sex for all temperatures with >60 years old being the predominant age group. The majority of patients were discharged within seven hours (<4 h: 32%-38%, 4–7 h: 33%-35%) with minor differences across temperature groups. A rise of 2.6% and 1.9% was observed on visits lasting 12–23 h and ≥24 h respectively as the temperature increased. The highest triage acuity was P3 following an inverted U-shaped curve with a peak at 29.5–31 °C (46.5%), and a 3% decrease at both temperature extremes. Mortality fluctuated between 2.9% to 4.4% across temperature groups. At ≥31 °C, the mortality rate was the highest (4.4%) compared to the other NCDs in the study.

#### 3.4.5 Cerebral vascular accident (CVA)

Males presented the most (51%-55%) across all temperature groups. The majority (51%-56%) of the visits were by patients >60 years. The proportion of patients discharged within four hours was uniformly high (>53%) across all temperature groups while <5% patients were discharged after 24 h. The prevalent triage acuity was P3 following an inverted U-shaped trend, followed by P2, P1 and P4/5. All-cause mortality had a peak of 4.1% at ≥31 °C with a U-shaped trend.

### 3.5 Patients’ visit and mortality rates of NCDs by mean daily ambient temperature

A gradual decrease in visit rate for each NCD as temperature increases was observed. (Fig. 2)

**Fig. 2:**
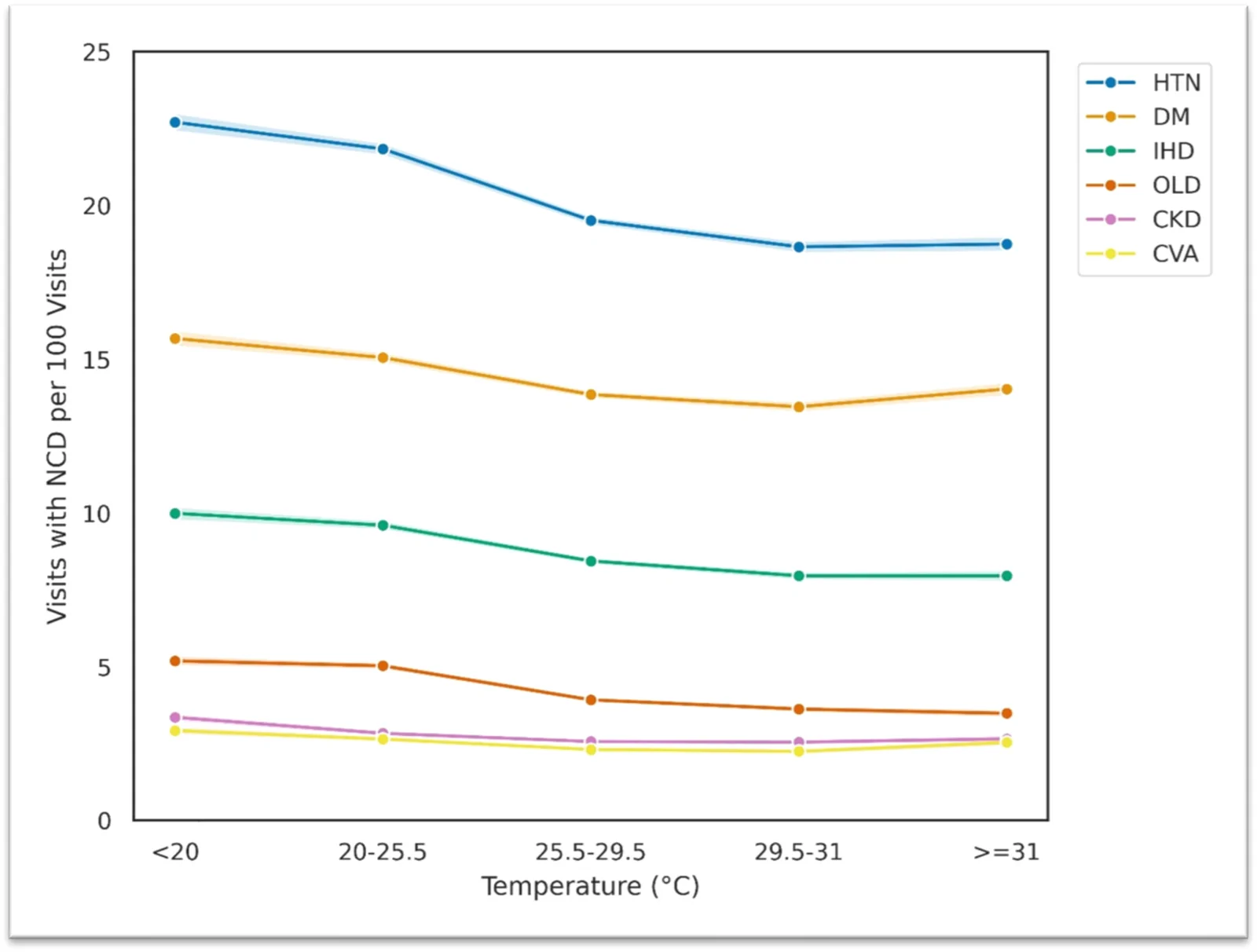
Stratification of non-communicable disease patient visits by mean daily ambient temperature. HTN: Hypertension, DM: Diabetes Mellitus, IHD: Ischemic Heart Disease, OLD: Obstructive Lung Disease, CKD: Chronic Kidney Disease, CVA: Cerebrovascular Accident.

Most NCDs followed a U-shaped curve for mortality rate. Patients with CKD had a higher mortality rate overall (2.9%-4.5%) when compared to other NCDs. Patients with CVA showed a gradual increase in mortality rate as temperature rose, with a sharp rise in temperatures ≥40 °C (Fig. 3).

**Fig. 3:**
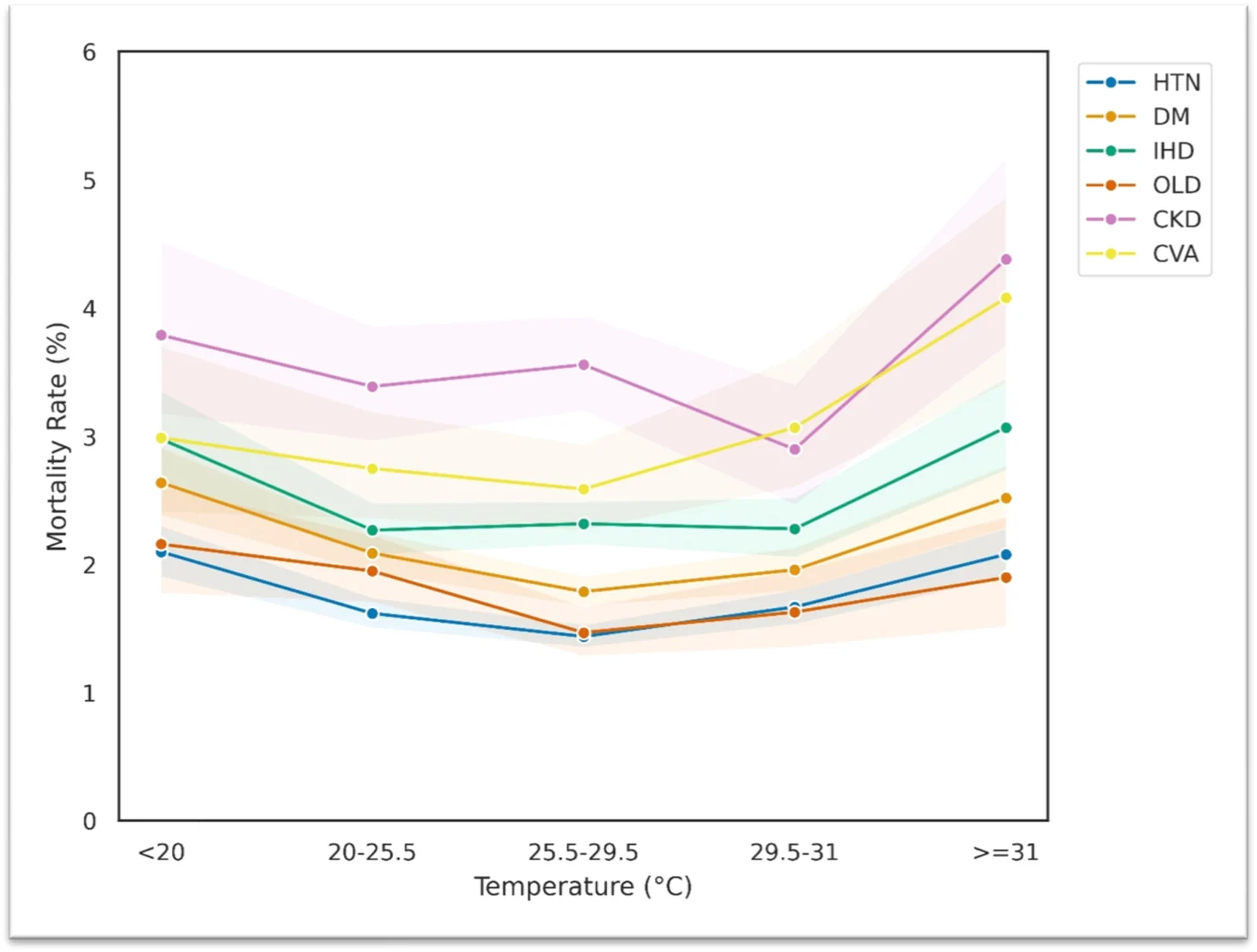
Stratification of non-communicable disease patient mortality rate with mean daily ambient temperature and 95% confidence intervals. HTN: Hypertension, DM: Diabetes Mellitus, IHD: Ischemic Heart Disease, OLD: Obstructive Lung Disease, CKD: Chronic Kidney Disease, CVA: Cerebrovascular Accident. Shaded portions of the figure represent the 95% confidence intervals.

### 3.6 Time to in-hospital death for NCDs by mean daily ambient temperature

Using a Weibull AFT model, the effect of ambient temperature on NCD median survival time was estimated after adjusting for age, sex, and triage acuity. For mean daily ambient temperature, the reference category was temperature group 25.5–29.5 °C. One model was built for each NCD (Fig. 4, Appendix 5).

**Fig. 4:**
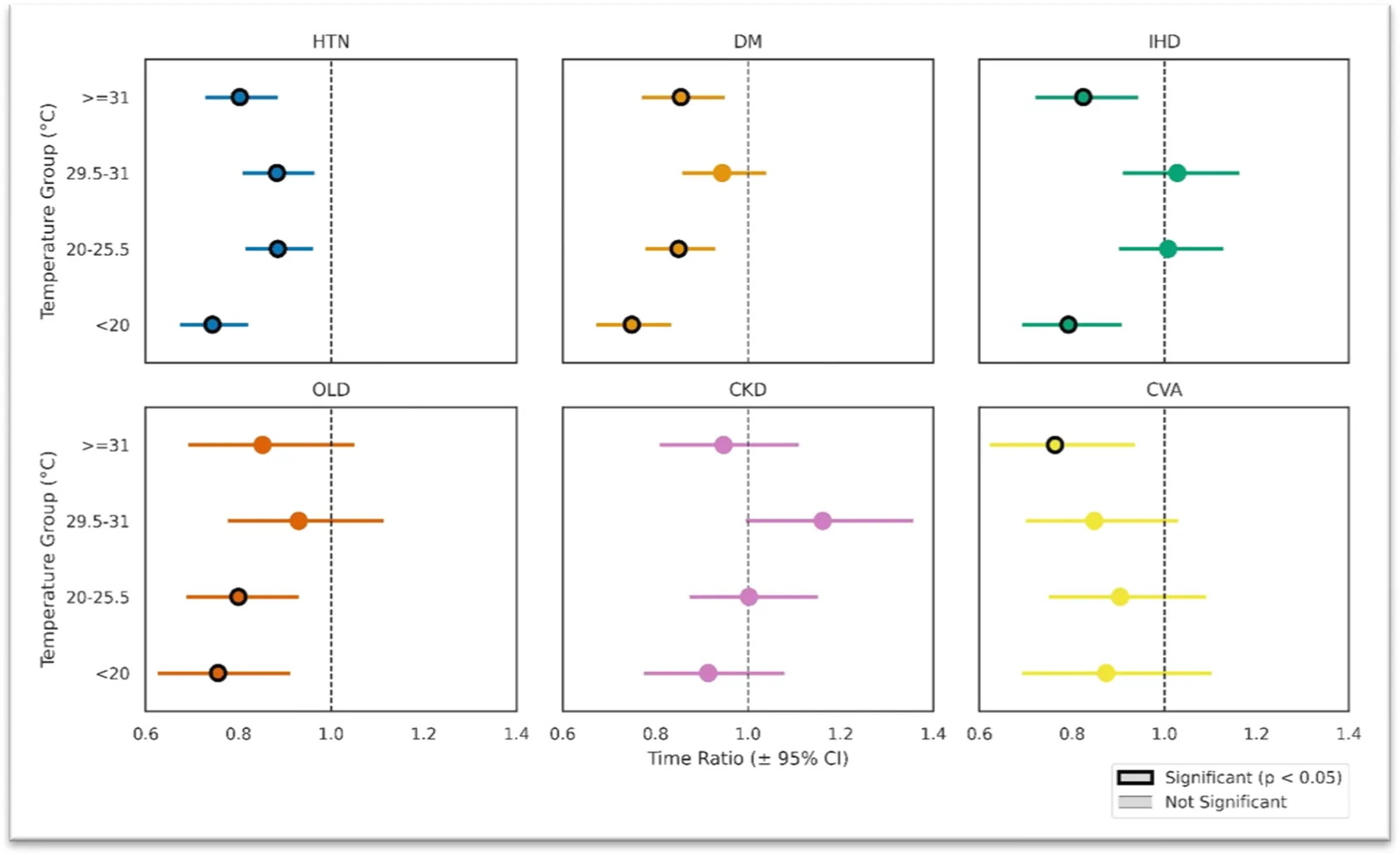
Accelerated Failure Time (AFT) models assessing the relationship between non-communicable disease survival time and mean daily ambient temperature (adjusted for age, sex, and triage acuity with 95% CI). Reference category is 25.5-29.5 °C. HTN: Hypertension, DM: Diabetes Mellitus, IHD: Ischemic Heart Disease, OLD: Obstructive Lung Disease, CKD: Chronic Kidney Disease, CVA: Cerebrovascular Accident.

#### 3.6.1 Hypertension (HTN)

The concordance (C-index) of the adjusted AFT model for HTN was 0.82, which indicated good predictive ability. The median survival time was 4.35 h (IQR: 2.50–7.13). All temperature groups were independently associated with significantly shorter time to death when compared to the reference range of 25.5–29.5 °C. The lowest temperatures (<20 °C) corresponded to 26% acceleration to death (TR: 0.74, 95% CI 0.67–0.82), while the highest temperatures (≥31 °C) corresponded to 20% acceleration (TR: 0.80, 95% CI 0.73–0.89) compared to the reference group. The middle temperature groups (20.5–25.5 °C and 29.5–31 °C) corresponded to 12% (TR: 0.88, 95% CI 0.81–0.96) and 11% (TR: 0.89, 95% CI 0.82–0.96) acceleration to death respectively.

#### 3.6.2 Diabetes mellitus (DM)

DM visits had a median survival time of 4.45 h (IQR: 2.55–7.35). The model (C-index: 0.82) confirmed temperatures <20 °C had the most significant association with time to death, corresponding to a 25% (TR: 0.75, 95% CI 0.67–0.83) acceleration to death compared to reference group 25.5–29.5°C. Temperature groups 20–25.5 °C and ≥31 °C saw similarly significant patterns, with 15% (TR: 0.85, 95% CI 0.78–0.93) and 14% (TR: 0.86, 95% CI 0.77–0.95) earlier deaths respectively.

#### 3.6.3 Ischemic heart disease (IHD)

IHD had a median survival time of 4.55 h (IQR: 2.69–7.61). The adjusted model found significant associations with both extreme temperature groups (<20 °C and ≥31 °C). At <20 °C, the time to death was 21% (TR: 0.79, 95% CI 0.69–0.91) earlier than the reference group (25.5–29.5 °C) while at ≥31 °C it was 18% (TR: 0.82, 95% CI 0.72–0.94) earlier.

#### 3.6.4 Obstructive lung disease (OLD)

The median survival time for OLD visits was 4.32 h (IQR: 2.55–7.00). The adjusted Accelerated Failure Time model (C-Index: 0.77) only found significant associations for the lower temperature groups (<20 °C and 20–25.5 °C) when compared to the reference group (25.5–29.5 °C). At <20 °C, the time to death was 24% (TR: 0.76, 95% CI 0.63–0.91) earlier than the reference temperature group (25.5–29.5 °C) while at 20–25.5 °C it was 20% (TR: 0.80, 95% CI 0.69–0.93) earlier.

#### 3.6.5 Chronic kidney disease (CKD)

CKD visits observed a median survival time of 5.39 h (IQR: 3.17–9.28). The adjusted model (C-index: 0.75) found that warm temperatures (29.5–31 °C) experienced a 5% (TR: 0.95, 95% CI 0.81–1.11) acceleration in time to death relative to the reference range of 25.5–29.5 °C.

#### 3.6.6 Cerebrovascular accident (CVA)

Median survival time of CVA visits was 3.48 h (IQR: 1.53–6.43). In multivariable Accelerated Failure Time regression (C-index: 0.77), temperatures ≥31 °C were associated with a 24% (TR: 0.76, 95% CI 0.62–0.94) acceleration in time to death versus the reference category of 25.5–29.5 °C.

### 3.7 Effect of extreme temperature (≥31 °C) lag upon NCD mortality

Although some differences in lag effect of temperature on mortality rate were noted visually (Appendix 6) for all temperature groups, the most notable differences were in the temperature groups <20 °C and ≥31 °C (Fig. 5). This section focuses on lag effect of extreme mean daily temperatures (≥31 °C) on mortality rate using Autoregressive Distributed Lag for each NCD (Appendix 7).

**Fig. 5:**
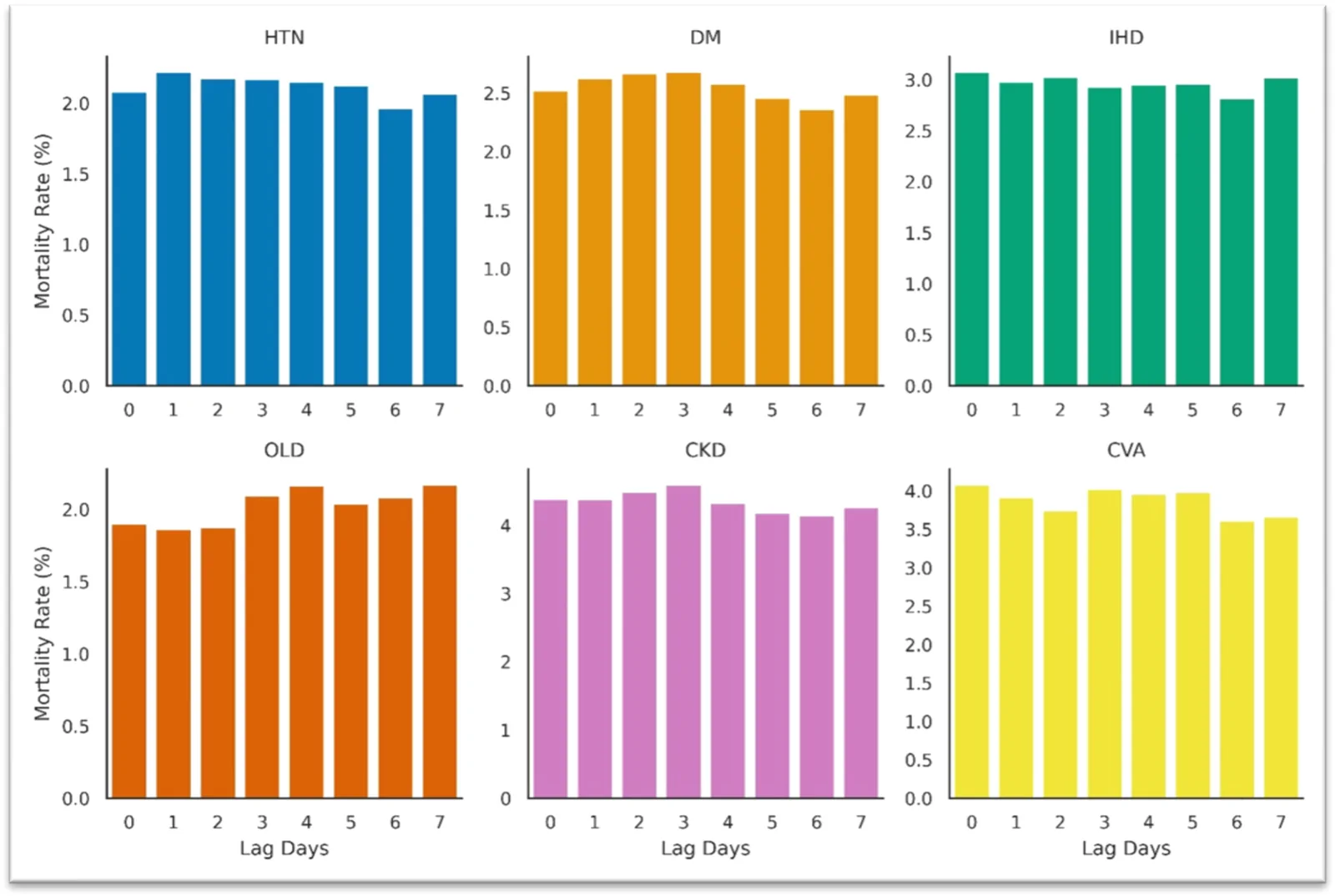
Mortality rate of non-communicable diseases at ≥31 °C with 7 days temperature lag. HTN: Hypertension, DM: Diabetes Mellitus, IHD: Ischemic Heart Disease, OLD: Obstructive Lung Disease, CKD: Chronic Kidney Disease, CVA: Cerebrovascular Accident.

For HTN patients on one day after exposure (lag 1) to extreme mean daily temperature (≥31 °C), mortality rate was increased (2.22%) from day 0 (2.22%). This declined to 1.96% at day 6 and returned to original by day 7. A significant positive association was observed at lag day 1 (β = 0.49, 95% CI: 0.10–0.89), indicating slightly delayed effects of heat exposure.

DM saw increasing mortality rates until lag day 3, followed by a gradual decrease over the next four days to lower mortality rates.

IHD experienced fluctuations in mortality rates throughout the week after exposure to extreme temperatures. Mortality rate showed a significant association with same-day extreme temperature (≥31 °C) (β = 0.74, 95% CI: 0.19–1.30), as did lag day 7 (β = 0.56, 95% CI: 0.00–1.11), highlighting an immediate and delayed response to extreme heat in this group.

OLD experienced two peaks in mortality during the week following exposure to extreme mean daily temperature (≥31 °C) at lag day 4 (2.16%) and day 7 (2.17%)

CKD patients, however, only experienced one peak in mortality on lag day 3, increasing from 4.38% to 4.59%. CVA experienced decreased mortality with lags, except for lag day 3 when the mortality rate returned to the same as lag day 0 (4.03%). For CVA, a strong delayed increased response was noted at lag day 5 (β = 2.20, 95% CI: 0.59–3.81). Overall, the results indicated that extreme heat events were associated with a delayed increase in mortality rate, particularly among patients with HTN, IHD, and CVA (Fig. 6, Appendix 7).

**Fig. 6:**
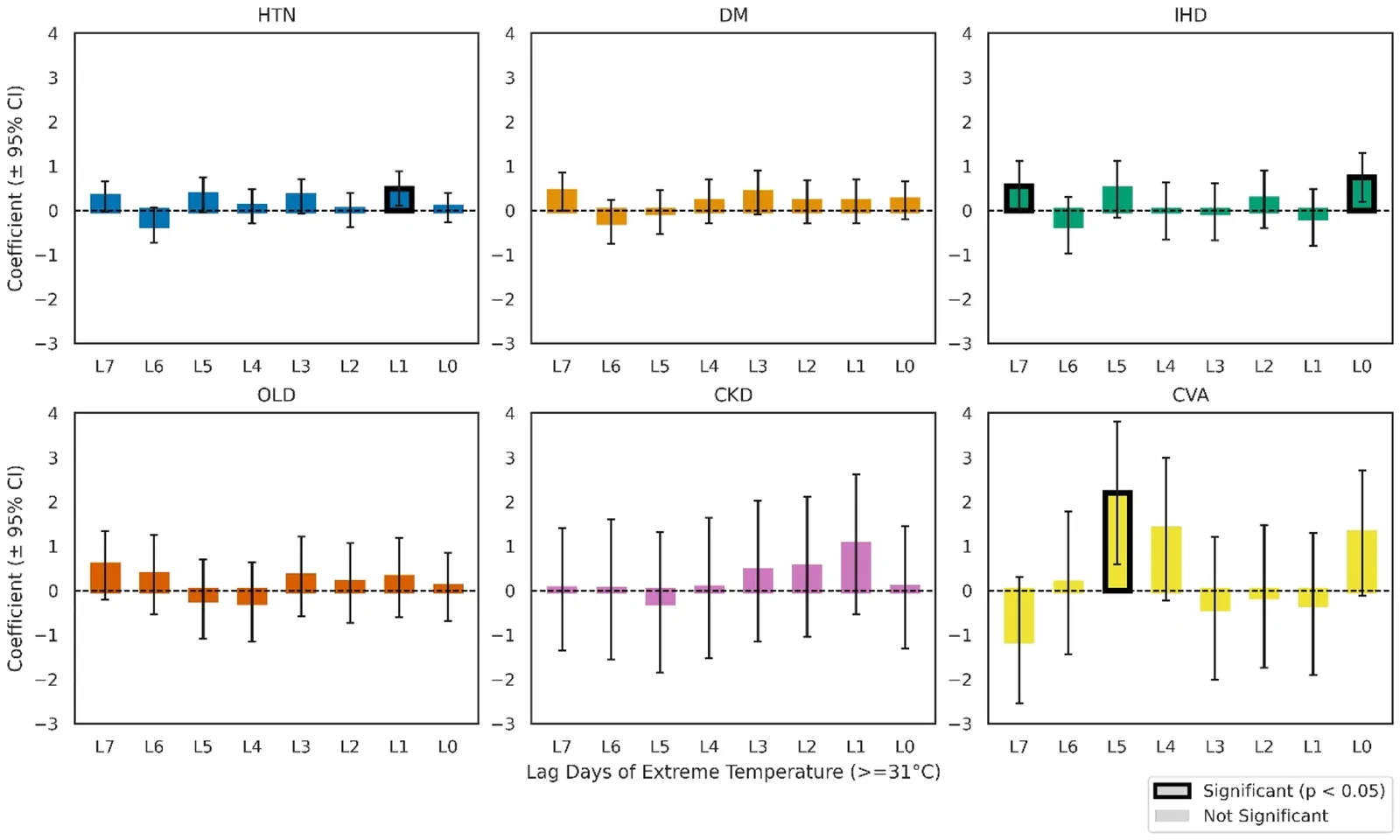
Relationship of non-communicable disease 7 lag day mortality and extreme daily ambient temperature established through Autoregressive Distributed Lag model. HTN: Hypertension, DM: Diabetes Mellitus, IHD: Ischemic Heart Disease, OLD: Obstructive Lung Disease, CKD: Chronic Kidney Disease, CVA: Cerebrovascular Accident.

Estimated coefficients with 95% confidence intervals for each lag (L0-L7) along with the constant term. Significant effects (p < 0.05) are indicated with bold borders

## 4 Discussion

### 4.1 Limitations

This study was conducted at a single center in Karachi, Pakistan which may limit the generalizability of the results to other geographic regions that experience different temperature patterns. However, the increased NCD mortality at extremes in temperature seen in this study mirrors trends in the global literature. This suggests that although climates vary across Pakistan and the Asian continent, general trends may be applicable to other climates.

Ambient temperature was used as a proxy for heat exposure in our study, but it may not directly equate to the temperature experienced by all individuals. The ambient daily mean temperature does not account for the time individuals were outdoors versus indoors. Central heat and air conditioning are uncommon in Karachi, so the impact of this limitation may be less substantial than in contexts where indoor heating and cooling systems are common or mandated.

The data were collected in this study via a retrospective chart review. Like other retrospective studies, our study may be subject to selection bias. IHHN provides care to ED patients with many NCDs, not only those reported in this study. NCD-related ED visits that did not meet our inclusion criteria, such as chronic liver disease or cancer, may have trends that differ from those studied. Although we explored the influence of temperature on six specific NCDs, there may be a potential compounding effect of a patient with multiple NCDs simultaneously that may impact the NCD mortality rate results.

Lastly, there are other temperature-dependent variables to consider that may confound the findings of this study. Some considerations include infectious diseases, such as respiratory infections causing obstructive lung disease flares, or viral infections triggering diabetic emergencies. Temperature-related fires, electrical outages, floods, and other natural disasters can impact supply chain integrity, pharmaceutical availability, transportation reliability, and other factors that may impact NCD outcomes.

### 4.2 In-depth discussion

This decade long analysis of over one million ED visits from a major urban center in Pakistan reveals a clear association between ambient temperature and patterns of NCD. The results of our study reinforce the growing understanding that climate extremes exert a measurable and clinically important influence on NCD presentation and mortality. These findings have implications on emergency care systems, public health, and disaster preparedness, especially in regions like Pakistan that are disproportionally impacted by climate change [12,22,23].

Consistent with global data, it was observed that colder temperatures were associated with increased NCD-related ED visits and higher mortality across most NCD categories, including HTN, DM, IHD, OLD, and CKD [[5], [6], [7], [8], [9], [10], [11], [12], [13], [14], [15], [16], [17], [18], [19], [20], [21],^31^,^32^]. While the physiological impact of cold, such as increased vasoconstriction, sympathetic activity and inflammatory markers is well documented [7,10,11,33,34], its implications in a resource-limited country, like Pakistan, remain underappreciated. This requires an in-depth understanding especially because climate change is not only a threat to the environment, but also to the economic security of Pakistan [35].

CVA mortality followed a contrasting pattern, with higher deaths occurring during warmer temperatures. Though traditionally associated with cold weather risk [[7], [8], [9]], emerging evidence has shown that heat waves and diurnal heat variability increase the risk of CVA, particularly among elderly individuals with dehydration, electrolyte disturbances, or pre-existing vascular fragility [36,37]. The delayed mortality observed in the study cohort following extreme heat events, especially in patients with HTN, IHD, and CVA, highlights the insidious effect of sustained high temperatures on physiological reserve. These findings support prior work from East Asia and the Middle East demonstrating lagged increases in cardiovascular and respiratory deaths following periods of extreme heat [38,39].

Pakistan ranks amongst the topmost climate-impacted countries globally, facing record-breaking heat waves, monsoon variability and urban flooding, all of which exacerbate chronic disease vulnerability [12,22,23]. The city of Karachi has witnessed flash flooding and heat waves in recent years, with temperatures crossing 49 °C and leading to hundreds of deaths, overwhelming EDs already strained by resource limitations [40]. Similar concerns were voiced in literature from India where a need was felt for better NCD surveillance in a changing urban climate [41]. Against this backdrop, the findings of this study emphasize the need for climate-informed recommendations to improve emergency care systems, public health, and disaster preparedness.

First, emergency care system operations can be streamlined to be more seasonally adaptive and based on temperature-related trends in ED visits and disease mortality. The increase in NCD-related ED visits during colder months and the lagged mortality in extreme heat provides an opportunity to tailor staffing rosters, hospital bed availability, and medication and supply stockpiles to these trends. Optimizing emergency care operations would promote improved quality and continuity of care for patients with NCDs. If resources are optimized to when and where they are most needed, these interventions may have a cost-effective benefit. These changes in ED operations should be based on continued surveillance data and be followed with future studies to confirm their impact on disease mortality.

Second, more climate-informed public health infrastructure is needed in resource-limited countries, like Pakistan, affected by climate change and extreme weather events. As shown in multiple other studies globally, our study reiterates a clear connection between temperature extremes and poor outcomes associated with multiple NCDs [[5], [6], [7], [8], [9], [10], [11], [12], [13], [14], [15], [16], [17], [18], [19], [20], [21], [22], [23], [24], [25], [26]]. Limited access to indoor heating or cooling systems in Pakistan’s lower-income communities may exacerbate the physiologic toll of temperature extremes. Additionally, vulnerable populations, like the elderly, children, and individuals of low socioeconomic status may be at particular risk [11,13]. While indoor temperature control is a protective factor in higher income countries, its absence in most Pakistani households renders ambient temperature a far more direct determinant of health outcomes [42]. In such settings, EDs serve not only as sites for acute care but also as critical safety nets during environmental crises. This highlights the importance of more cooling and heating centers, hydration stations during heat waves, and improved housing infrastructure.

Third, the climate-health connection also extends to emergency preparedness. The lag in mortality in HTN, IHD, and CVA patients after heat exposure suggests that traditional surveillance may miss preventable outcomes. Community-based early warning systems, mobile health units, and targeted messaging to high-risk populations could minimize downstream ED visits and deaths. Integration of temperature forecasts into clinical risk scoring tools may also aid in triage and follow-up planning during seasonal transitions [43,44].

## 5 Conclusion

These results demonstrate that both low and high ambient temperatures significantly impact NCD-related ED presentations and outcomes. Cold weather is associated with higher visit rates and mortality for most NCDs, while extreme heat results in delayed mortality, particularly among patients with CVA, IHD, and HTN. These findings carry urgent implications for clinical operations, public health, and emergency preparedness and climate resilience in a low-resource setting like Pakistan. EDs must evolve beyond reactive care models towards proactive, seasonally responsive care. Future directions should include multi-center studies incorporating granular exposure data and pilot programs integrating meteorological data into real-time triage and chronic disease management pathways.

## Funding

This work has not received any funding or financial support in its investigation or manuscript development.

## CRediT authorship contribution statement

**Syed Ghazanfar Saleem:** Supervision, Data curation, Conceptualization. **Zainab Tariq Soomro:** Writing – review & editing, Writing – original draft, Methodology, Formal analysis. **Saima Ali:** Writing – review & editing, Writing – original draft, Supervision, Conceptualization. **Ammad Fahim:** Conceptualization. **Megan Rybarczyk:** Writing – review & editing. **Joseph Dominick Ciano:** Writing – review & editing, Writing – original draft, Supervision, Project administration, Conceptualization.

## Declaration of competing interest

The authors declare the following financial interests/personal relationships which may be considered as potential competing interests:

Joseph Ciano reports statistical analysis and writing assistance were provided by The Indus Hospital Korangi Campus. Joseph Ciano reports a relationship with Hospital of the University of Pennsylvania that includes: employment and non-financial support. Other authors declare that they have no known competing financial interests or personal relationships that could have appeared to influence the work reported in this paper.
