# Supplementary material for: Ambient temperature and non-communicable disease presentations and mortality in a Pakistani ED
Source: J Clim Chang Health. 2026 Oct 28;32:100768. doi: 10.1016/j.joclim.2026.100768 (PMC13742321; doi:10.1016/j.joclim.2026.100768)
Supplement: Supplementary file 1 [file mmc1.docx]

Appendix 1. Inclusion and exclusion criteria for the study

Appendix 2. Presentation of non-communicable disease in adult emergency department.
**HTN: Hypertension, DM: Diabetes Mellitus, IHD: Ischemic Heart Disease, OLD: Obstructive Lung Disease, CKD: Chronic Kidney Disease, CVA: Cerebrovascular Accident*

| HTN | DM | IHD | OLD | CKD | CVA | Visits |
| --- | --- | --- | --- | --- | --- | --- |
| FALSE | FALSE | FALSE | FALSE | FALSE | FALSE | 700,202 |
| TRUE | FALSE | FALSE | FALSE | FALSE | FALSE | 69,324 |
| FALSE | TRUE | FALSE | FALSE | FALSE | FALSE | 41,820 |
| TRUE | TRUE | FALSE | FALSE | FALSE | FALSE | 40,031 |
| TRUE | TRUE | TRUE | FALSE | FALSE | FALSE | 24,310 |
| FALSE | FALSE | FALSE | TRUE | FALSE | FALSE | 23,470 |
| TRUE | FALSE | TRUE | FALSE | FALSE | FALSE | 19,925 |
| FALSE | FALSE | TRUE | FALSE | FALSE | FALSE | 15,038 |
| TRUE | TRUE | TRUE | FALSE | TRUE | FALSE | 6,153 |
| TRUE | FALSE | FALSE | FALSE | FALSE | TRUE | 5,424 |
| TRUE | FALSE | FALSE | TRUE | FALSE | FALSE | 5,339 |
| FALSE | TRUE | TRUE | FALSE | FALSE | FALSE | 5,268 |
| TRUE | TRUE | FALSE | FALSE | TRUE | FALSE | 4,731 |
| FALSE | FALSE | FALSE | FALSE | FALSE | TRUE | 4,490 |
| TRUE | TRUE | FALSE | FALSE | FALSE | TRUE | 4,452 |
| FALSE | FALSE | FALSE | FALSE | TRUE | FALSE | 4,432 |
| TRUE | FALSE | FALSE | FALSE | TRUE | FALSE | 3,719 |
| TRUE | TRUE | TRUE | FALSE | FALSE | TRUE | 3,095 |
| TRUE | TRUE | FALSE | TRUE | FALSE | FALSE | 2,881 |
| TRUE | TRUE | TRUE | TRUE | FALSE | FALSE | 2,090 |
| TRUE | FALSE | TRUE | TRUE | FALSE | FALSE | 2,041 |
| TRUE | FALSE | TRUE | FALSE | FALSE | TRUE | 1,732 |
| TRUE | FALSE | TRUE | FALSE | TRUE | FALSE | 1,570 |
| FALSE | TRUE | FALSE | FALSE | TRUE | FALSE | 1,428 |
| FALSE | TRUE | FALSE | TRUE | FALSE | FALSE | 1,374 |
| FALSE | FALSE | TRUE | TRUE | FALSE | FALSE | 1,310 |
| FALSE | TRUE | FALSE | FALSE | FALSE | TRUE | 1,015 |
| TRUE | TRUE | TRUE | FALSE | TRUE | TRUE | 990 |
| TRUE | TRUE | TRUE | TRUE | TRUE | FALSE | 703 |
| TRUE | TRUE | FALSE | FALSE | TRUE | TRUE | 585 |
| FALSE | FALSE | TRUE | FALSE | TRUE | FALSE | 461 |
| FALSE | TRUE | TRUE | FALSE | TRUE | FALSE | 421 |
| FALSE | FALSE | TRUE | FALSE | FALSE | TRUE | 414 |
| TRUE | FALSE | FALSE | TRUE | FALSE | TRUE | 321 |
| TRUE | FALSE | FALSE | FALSE | TRUE | TRUE | 288 |
| TRUE | TRUE | TRUE | TRUE | TRUE | TRUE | 273 |
| TRUE | TRUE | TRUE | TRUE | FALSE | TRUE | 248 |
| FALSE | TRUE | TRUE | TRUE | FALSE | FALSE | 240 |
| FALSE | FALSE | FALSE | TRUE | TRUE | FALSE | 225 |
| FALSE | FALSE | FALSE | TRUE | FALSE | TRUE | 214 |
| FALSE | TRUE | TRUE | FALSE | FALSE | TRUE | 212 |
| TRUE | FALSE | TRUE | TRUE | TRUE | FALSE | 212 |
| TRUE | FALSE | TRUE | TRUE | FALSE | TRUE | 190 |
| TRUE | FALSE | TRUE | FALSE | TRUE | TRUE | 186 |
| TRUE | TRUE | FALSE | TRUE | TRUE | FALSE | 185 |
| TRUE | FALSE | FALSE | TRUE | TRUE | FALSE | 171 |
| TRUE | TRUE | FALSE | TRUE | FALSE | TRUE | 160 |
| FALSE | FALSE | FALSE | FALSE | TRUE | TRUE | 71 |
| FALSE | FALSE | TRUE | TRUE | TRUE | FALSE | 65 |
| FALSE | TRUE | FALSE | FALSE | TRUE | TRUE | 61 |
| TRUE | FALSE | TRUE | TRUE | TRUE | TRUE | 56 |
| TRUE | TRUE | FALSE | TRUE | TRUE | TRUE | 51 |
| FALSE | TRUE | FALSE | TRUE | TRUE | FALSE | 43 |
| TRUE | FALSE | FALSE | TRUE | TRUE | TRUE | 29 |
| FALSE | FALSE | TRUE | TRUE | FALSE | TRUE | 27 |
| FALSE | TRUE | TRUE | TRUE | TRUE | FALSE | 27 |
| FALSE | TRUE | TRUE | FALSE | TRUE | TRUE | 25 |
| FALSE | FALSE | TRUE | FALSE | TRUE | TRUE | 22 |
| FALSE | TRUE | FALSE | TRUE | FALSE | TRUE | 16 |
| FALSE | TRUE | TRUE | TRUE | FALSE | TRUE | 15 |
| FALSE | FALSE | FALSE | TRUE | TRUE | TRUE | 11 |
| FALSE | TRUE | FALSE | TRUE | TRUE | TRUE | 7 |
| FALSE | FALSE | TRUE | TRUE | TRUE | TRUE | 3 |
| FALSE | TRUE | TRUE | TRUE | TRUE | TRUE | 2 |
|  |  |  |  |  | **Total** | **1,003,894** |

Appendix 3. Repeated visits by patients with non-communicable disease in adult emergency department.

| Number of visits | Number of Patients |
| --- | --- |
| 1 | 455,216 |
| 2 | 86,572 |
| 3 | 31,621 |
| 4 | 15,262 |
| 5 | 8,444 |
| 6-10 | 13,949 |
| 11-20 | 3,780 |
| 21-50 | 749 |
| 51-100 | 43 |
| >100 | 7 |
| **Total Patients** | **615,643** |

Appendix 4. Demographic and clinical outcomes of visits by patients with non-communicable disease in adult emergency departments and their association with daily mean ambient temperature.
**HTN: Hypertension, DM: Diabetes Mellitus, IHD: Ischemic Heart Disease, OLD: Obstructive Lung Disease, CKD: Chronic Kidney Disease, CVA: Cerebrovascular Accident*

|  | N(%) | HTN | | | | | DM | | | | | IHD | | | | | OLD | | | | | CKD | | | | | CVA | | | | |
| --- | --- | --- | --- | --- | --- | --- | --- | --- | --- | --- | --- | --- | --- | --- | --- | --- | --- | --- | --- | --- | --- | --- | --- | --- | --- | --- | --- | --- | --- | --- | --- |
| Variable | Sub-category | <20 | 20-25.5 | 25.5-29.5 | 29.5-31 | ≥31 | <20 | 20-25.5 | 25.5-29.5 | 29.5-31 | ≥31 | <20 | 20-25.5 | 25.5-29.5 | 29.5-31 | ≥31 | <20 | 20-25.5 | 25.5-29.5 | 29.5-31 | ≥31 | <20 | 20-25.5 | 25.5-29.5 | 29.5-31 | ≥31 | <20 | 20-25.5 | 25.5-29.5 | 29.5-31 | ≥31 |
| Total visits | | 21043 | 48571 | 73049 | 37283 | 21519 | 14531 | 33504 | 51883 | 26885 | 16109 | 9262 | 21384 | 31622 | 15913 | 9143 | 4819 | 11206 | 14720 | 7250 | 4004 | 3110 | 6317 | 9619 | 5103 | 3057 | 2712 | 5900 | 8664 | 4492 | 2917 |
| Gender | Female | 12521 (59.5) | 29112 (59.9) | 44431 (60.8) | 22789 (61.1) | 13043 (60.6) | 8198 (56.4) | 18828 (56.2) | 29440 (56.7) | 15215 (56.6) | 8983 (55.8) | 3958 (42.7) | 9034 (42.2) | 13513 (42.7) | 6764 (42.5) | 3902 (42.7) | 2347 (48.7) | 5465 (48.8) | 7168 (48.7) | 3675 (50.7) | 1967 (49.1) | 1545 (49.7) | 3091 (48.9) | 4593 (47.7) | 2465 (48.3) | 1479 (48.4) | 1248 (46.0) | 2665 (45.2) | 4092 (47.2) | 2196 (48.9) | 1390 (47.7) |
|  | Male | 8522 (40.5) | 19459 (40.1) | 28618 (39.2) | 14494 (38.9) | 8476 (39.4) | 6333 (43.6) | 14676 (43.8) | 22443 (43.3) | 11670 (43.4) | 7126 (44.2) | 5304 (57.3) | 12350 (57.8) | 18109 (57.3) | 9149 (57.5) | 5241 (57.3) | 2472 (51.3) | 5741 (51.2) | 7552 (51.3) | 3575 (49.3) | 2037 (50.9) | 1565 (50.3) | 3226 (51.1) | 5026 (52.3) | 2638 (51.7) | 1578 (51.6) | 1464 (54.0) | 3235 (54.8) | 4572 (52.8) | 2296 (51.1) | 1527 (52.3) |
| Age (Years) | 18-29 | 519 (2.5) | 1197 (2.5) | 1932 (2.6) | 950 (2.5) | 586 (2.7) | 397 (2.7) | 913 (2.7) | 1451 (2.8) | 795 (3.0) | 451 (2.8) | 152 (1.6) | 341 (1.6) | 593 (1.9) | 264 (1.7) | 161 (1.8) | 591 (12.3) | 1438 (12.8) | 2165 (14.7) | 1008 (13.9) | 551 (13.8) | 168 (5.4) | 338 (5.4) | 534 (5.6) | 284 (5.6) | 180 (5.9) | 79 (2.9) | 193 (3.3) | 344 (4.0) | 160 (3.6) | 87 (3.0) |
|  | 30-39 | 1852 (8.8) | 4265 (8.8) | 6758 (9.3) | 3667 (9.8) | 2008 (9.3) | 990 (6.8) | 2306 (6.9) | 4016 (7.7) | 2143 (8.0) | 1286 (8.0) | 441 (4.8) | 1070 (5.0) | 1607 (5.1) | 902 (5.7) | 462 (5.1) | 671 (13.9) | 1562 (13.9) | 2286 (15.5) | 1217 (16.8) | 681 (17.0) | 228 (7.3) | 516 (8.2) | 852 (8.9) | 447 (8.8) | 277 (9.1) | 151 (5.6) | 323 (5.5) | 521 (6.0) | 259 (5.8) | 146 (5.0) |
|  | 40-49 | 4184 (19.9) | 9950 (20.5) | 15787 (21.6) | 7928 (21.3) | 4634 (21.5) | 2687 (18.5) | 6545 (19.5) | 10581 (20.4) | 5401 (20.1) | 3293 (20.4) | 1366 (14.7) | 3380 (15.8) | 5289 (16.7) | 2634 (16.6) | 1568 (17.1) | 735 (15.3) | 1741 (15.5) | 2383 (16.2) | 1156 (15.9) | 608 (15.2) | 478 (15.4) | 1034 (16.4) | 1541 (16.0) | 817 (16.0) | 489 (16.0) | 365 (13.5) | 764 (12.9) | 1233 (14.2) | 656 (14.6) | 397 (13.6) |
|  | 50-59 | 5784 (27.5) | 13460 (27.7) | 20471 (28.0) | 10569 (28.3) | 5772 (26.8) | 4423 (30.4) | 10268 (30.6) | 16037 (30.9) | 8456 (31.5) | 4924 (30.6) | 2685 (29.0) | 6208 (29.0) | 9450 (29.9) | 4819 (30.3) | 2622 (28.7) | 1013 (21.0) | 2228 (19.9) | 2758 (18.7) | 1402 (19.3) | 767 (19.2) | 852 (27.4) | 1650 (26.1) | 2591 (26.9) | 1412 (27.7) | 737 (24.1) | 661 (24.4) | 1425 (24.2) | 2097 (24.2) | 1067 (23.8) | 671 (23.0) |
|  | 60+ | 8704 (41.4) | 19699 (40.6) | 28101 (38.5) | 14169 (38.0) | 8519 (39.6) | 6034 (41.5) | 13472 (40.2) | 19798 (38.2) | 10090 (37.5) | 6155 (38.2) | 4618 (49.9) | 10385 (48.6) | 14683 (46.4) | 7294 (45.8) | 4330 (47.4) | 1809 (37.5) | 4237 (37.8) | 5128 (34.8) | 2467 (34.0) | 1397 (34.9) | 1384 (44.5) | 2779 (44.0) | 4101 (42.6) | 2143 (42.0) | 1374 (44.9) | 1456 (53.7) | 3195 (54.2) | 4469 (51.6) | 2350 (52.3) | 1616 (55.4) |
| Length of stay in hospital (hours) | <4 | 9979 (47.4) | 22947 (47.2) | 32474 (44.5) | 16777 (45.0) | 9449 (43.9) | 6645 (45.7) | 15524 (46.3) | 22599 (43.6) | 11805 (43.9) | 6896 (42.8) | 4163 (44.9) | 9557 (44.7) | 13383 (42.3) | 6760 (42.5) | 3759 (41.1) | 2239 (46.5) | 5376 (48.0) | 6577 (44.7) | 3223 (44.5) | 1806 (45.1) | 1140 (36.7) | 2354 (37.3) | 3192 (33.2) | 1750 (34.3) | 1002 (32.8) | 1615 (59.6) | 3392 (57.5) | 4792 (55.3) | 2487 (55.4) | 1547 (53.0) |
|  | 4-7 | 7033 (33.4) | 16378 (33.7) | 25451 (34.8) | 12691 (34.0) | 7189 (33.4) | 4881 (33.6) | 11160 (33.3) | 18020 (34.7) | 9181 (34.1) | 5299 (32.9) | 3072 (33.2) | 7138 (33.4) | 10820 (34.2) | 5343 (33.6) | 3042 (33.3) | 1651 (34.3) | 3835 (34.2) | 5194 (35.3) | 2613 (36.0) | 1339 (33.4) | 1085 (34.9) | 2162 (34.2) | 3361 (34.9) | 1735 (34.0) | 1026 (33.6) | 683 (25.2) | 1519 (25.7) | 2298 (26.5) | 1209 (26.9) | 782 (26.8) |
|  | 8-11 | 2041 (9.7) | 4853 (10.0) | 7962 (10.9) | 4114 (11.0) | 2385 (11.1) | 1485 (10.2) | 3532 (10.5) | 5781 (11.1) | 2936 (10.9) | 1812 (11.2) | 883 (9.5) | 2078 (9.7) | 3333 (10.5) | 1672 (10.5) | 1013 (11.1) | 513 (10.6) | 1046 (9.3) | 1587 (10.8) | 760 (10.5) | 441 (11.0) | 396 (12.7) | 746 (11.8) | 1314 (13.7) | 686 (13.4) | 409 (13.4) | 185 (6.8) | 493 (8.4) | 762 (8.8) | 400 (8.9) | 279 (9.6) |
|  | 12-23 | 1030 (4.9) | 2364 (4.9) | 3987 (5.5) | 2126 (5.7) | 1371 (6.4) | 797 (5.5) | 1777 (5.3) | 3040 (5.9) | 1686 (6.3) | 1135 (7.0) | 647 (7.0) | 1536 (7.2) | 2393 (7.6) | 1328 (8.3) | 752 (8.2) | 210 (4.4) | 490 (4.4) | 706 (4.8) | 345 (4.8) | 201 (5.0) | 267 (8.6) | 604 (9.6) | 1026 (10.7) | 542 (10.6) | 344 (11.3) | 114 (4.2) | 261 (4.4) | 435 (5.0) | 226 (5.0) | 171 (5.9) |
|  | ≥24 | 960 (4.6) | 2029 (4.2) | 3175 (4.3) | 1575 (4.2) | 1125 (5.2) | 723 (5.0) | 1511 (4.5) | 2443 (4.7) | 1277 (4.7) | 967 (6.0) | 497 (5.4) | 1075 (5.0) | 1693 (5.4) | 810 (5.1) | 577 (6.3) | 206 (4.3) | 459 (4.1) | 656 (4.5) | 309 (4.3) | 217 (5.4) | 222 (7.1) | 451 (7.1) | 726 (7.5) | 390 (7.6) | 276 (9.0) | 115 (4.2) | 235 (4.0) | 377 (4.4) | 170 (3.8) | 138 (4.7) |
| Triage Acuity | P1 | 2078 (9.9) | 4137 (8.5) | 5445 (7.5) | 2838 (7.6) | 2016 (9.4) | 1609 (11.1) | 3241 (9.7) | 4414 (8.5) | 2326 (8.7) | 1711 (10.6) | 1056 (11.4) | 2041 (9.5) | 2611 (8.3) | 1358 (8.5) | 884 (9.7) | 1083 (22.5) | 2503 (22.3) | 2665 (18.1) | 1285 (17.7) | 707 (17.7) | 446 (14.3) | 859 (13.6) | 1193 (12.4) | 623 (12.2) | 426 (13.9) | 543 (20.0) | 1060 (18.0) | 1404 (16.2) | 777 (17.3) | 638 (21.9) |
|  | P2 | 7104 (33.8) | 16791 (34.6) | 25501 (34.9) | 12561 (33.7) | 7217 (33.5) | 4815 (33.1) | 10990 (32.8) | 17466 (33.7) | 8869 (33.0) | 5223 (32.4) | 4118 (44.5) | 9729 (45.5) | 14710 (46.5) | 7199 (45.2) | 4016 (43.9) | 1926 (40.0) | 4528 (40.4) | 6198 (42.1) | 3015 (41.6) | 1595 (39.8) | 1176 (37.8) | 2382 (37.7) | 3714 (38.6) | 1856 (36.4) | 1174 (38.4) | 978 (36.1) | 2100 (35.6) | 3174 (36.6) | 1614 (35.9) | 1002 (34.4) |
|  | P3 | 10195 (48.4) | 24153 (49.7) | 37229 (51.0) | 19353 (51.9) | 10913 (50.7) | 7055 (48.6) | 16769 (50.1) | 26558 (51.2) | 13878 (51.6) | 8191 (50.8) | 3537 (38.2) | 8511 (39.8) | 12794 (40.5) | 6640 (41.7) | 3830 (41.9) | 1617 (33.6) | 3714 (33.1) | 5165 (35.1) | 2646 (36.5) | 1530 (38.2) | 1311 (42.2) | 2733 (43.3) | 4244 (44.1) | 2371 (46.5) | 1313 (43.0) | 1053 (38.8) | 2381 (40.4) | 3657 (42.2) | 1904 (42.4) | 1154 (39.6) |
|  | P4/P5 | 1666 (7.9) | 3490 (7.2) | 4874 (6.7) | 2531 (6.8) | 1373 (6.4) | 1052 (7.2) | 2504 (7.5) | 3445 (6.6) | 1812 (6.7) | 984 (6.1) | 551 (5.9) | 1103 (5.2) | 1507 (4.8) | 716 (4.5) | 413 (4.5) | 193 (4.0) | 461 (4.1) | 692 (4.7) | 304 (4.2) | 172 (4.3) | 177 (5.7) | 343 (5.4) | 468 (4.9) | 253 (5.0) | 144 (4.7) | 138 (5.1) | 359 (6.1) | 429 (5.0) | 197 (4.4) | 123 (4.2) |
| All cause mortality | No | 20602 (97.9) | 47783 (98.4) | 71994 (98.6) | 36662 (98.3) | 21071 (97.9) | 14148 (97.4) | 32804 (97.9) | 50955 (98.2) | 26359 (98.0) | 15703 (97.5) | 8986 (97.0) | 20899 (97.7) | 30889 (97.7) | 15550 (97.7) | 8862 (96.9) | 4715 (97.8) | 10987 (98.0) | 14504 (98.5) | 7132 (98.4) | 3928 (98.1) | 2992 (96.2) | 6103 (96.6) | 9277 (96.4) | 4955 (97.1) | 2923 (95.6) | 2631 (97.0) | 5738 (97.3) | 8440 (97.4) | 4354 (96.9) | 2798 (95.9) |
|  | Yes | 441 (2.1) | 788 (1.6) | 1055 (1.4) | 621 (1.7) | 448 (2.1) | 383 (2.6) | 700 (2.1) | 928 (1.8) | 526 (2.0) | 406 (2.5) | 276 (3.0) | 485 (2.3) | 733 (2.3) | 363 (2.3) | 281 (3.1) | 104 (2.2) | 219 (2.0) | 216 (1.5) | 118 (1.6) | 76 (1.9) | 118 (3.8) | 214 (3.4) | 342 (3.6) | 148 (2.9) | 134 (4.4) | 81 (3.0) | 162 (2.7) | 224 (2.6) | 138 (3.1) | 119 (4.1) |

Appendix 5. Estimated effects from Accelerated Failure Time (AFT) model assessing the relationship between temperature and emergency department survival time, adjusted for sex, acuity, and age in a study about adult emergency departments and their association with daily mean ambient temperature.
**HTN: Hypertension, DM: Diabetes Mellitus, IHD: Ischemic Heart Disease, OLD: Obstructive Lung Disease, CKD: Chronic Kidney Disease, CVA: Cerebrovascular Accident*

|  |  | HTN | | | | DM | | | | IHD | | | | OLD | | | | CKD | | | | CVA | | | |
| --- | --- | --- | --- | --- | --- | --- | --- | --- | --- | --- | --- | --- | --- | --- | --- | --- | --- | --- | --- | --- | --- | --- | --- | --- | --- |
| C Index | | 0.83 |  |  |  | 0.82 |  |  |  | 0.77 |  |  |  | 0.77 |  |  |  | 0.75 |  |  |  | 0.77 |  |  |  |
| Events | | 3,353 |  |  |  | 2,943 |  |  |  | 2,138 |  |  |  | 733 |  |  |  | 956 |  |  |  | 724 |  |  |  |
| Total visits | | 201,465 |  |  |  | 142,912 |  |  |  | 87,324 |  |  |  | 41,999 |  |  |  | 27,206 |  |  |  | 24,685 |  |  |  |
| Median Survival Time in Hours(IQR) | | 4.35 (2.50-7.13) |  |  |  | 4.45 (2.55-7.35) |  |  |  | 4.55 (2.69-7.61) |  |  |  | 4.32 (2.55-7.00) |  |  |  | 5.39 (3.17-9.28) |  |  |  | 3.48 (1.53-6.43) |  |  |  |
| Covariates | | Time Ratio | CI Lower | CI Upper | p-value | Time Ratio | CI Lower | CI Upper | p-value | Time Ratio | CI Lower | CI Upper | p-value | Time Ratio | CI Lower | CI Upper | p-value | Time Ratio | CI Lower | CI Upper | p-value | Time Ratio | CI Lower | CI Upper | p-value |
| Rho Intercept | | 1.11 | 1.08 | 1.15 | <0.001 | 1.10 | 1.07 | 1.14 | <0.001 | 1.01 | 0.98 | 1.05 | 0.480 | 1.23 | 1.15 | 1.31 | <0.001 | 1.22 | 1.15 | 1.30 | <0.001 | 1.07 | 1.01 | 1.14 | 0.020 |
| Age (Years) | | 0.98 | 0.97 | 0.98 | <0.001 | 0.98 | 0.98 | 0.98 | <0.001 | 0.97 | 0.97 | 0.97 | <0.001 | 0.98 | 0.97 | 0.98 | <0.001 | 0.99 | 0.99 | 0.99 | <0.001 | 0.99 | 0.98 | 0.99 | <0.001 |
| Sex | Female | 1.23 | 1.15 | 1.31 | <0.001 | 1.23 | 1.15 | 1.31 | <0.001 | 1.17 | 1.07 | 1.27 | <0.001 | 1.25 | 1.11 | 1.42 | <0.001 | 1.16 | 1.04 | 1.28 | 0.01 | 1.27 | 1.11 | 1.46 | 0.001 |
|  | Male (Ref.) | - | - | - | - | - | - | - | - | - | - | - | - | - | - | - | - | - | - | - | - | - | - | - | - |
| Triage Acuity | P1/P2 | 0.04 | 0.02 | 0.06 | <0.001 | 0.03 | 0.01 | 0.05 | <0.001 | 0.05 | 0.02 | 0.10 | <0.001 | 0 | 0 | 0 | <0.001 | 0.06 | 0.02 | 0.18 | - | 0.02 | 0.00 | 0.13 | - |
|  | P3 | 0.36 | 0.20 | 0.66 | <0.001 | 0.29 | 0.14 | 0.60 | 0.001 | 0.49 | 0.23 | 1.02 | 0.058 | 0 | 0 | 0 | <0.001 | 0.30 | 0.10 | 0.95 | 0.04 | 0.22 | 0.04 | 1.40 | 0.110 |
|  | P4/P5 (Ref.) | - | - | - | - | - | - | - | - | - | - | - | - | - | - | - | - | - | - | - | - | - | - | - | - |
| Mean Daily Ambient Temperature | <20 | 0.74 | 0.67 | 0.82 | <0.001 | 0.75 | 0.67 | 0.83 | <0.001 | 0.79 | 0.69 | 0.91 | 0.001 | 0.76 | 0.63 | 0.91 | <0.001 | 0.91 | 0.78 | 1.08 | 0.29 | 0.87 | 0.69 | 1.10 | 0.255 |
|  | 20-25.5 | 0.89 | 0.82 | 0.96 | <0.001 | 0.85 | 0.78 | 0.93 | <0.001 | 1.01 | 0.90 | 1.13 | 0.885 | 0.80 | 0.69 | 0.93 | <0.001 | 1.00 | 0.87 | 1.15 | 0.97 | 0.90 | 0.75 | 1.09 | 0.290 |
|  | 25.5-29.5 (Ref.) | - | - | - | - | - | - | - | - | - | - | - | - | - | - | - | - | - | - | - | - | - | - | - | - |
|  | 29.5-31 | 0.88 | 0.81 | 0.96 | 0.006 | 0.94 | 0.86 | 1.04 | 0.238 | 1.03 | 0.91 | 1.16 | 0.664 | 0.93 | 0.78 | 1.11 | 0.426 | 1.16 | 0.99 | 1.36 | 0.06 | 0.85 | 0.70 | 1.03 | 0.097 |
|  | >=31 | 0.80 | 0.73 | 0.89 | <0.001 | 0.86 | 0.77 | 0.95 | 0.003 | 0.82 | 0.72 | 0.94 | 0.005 | 0.85 | 0.69 | 1.05 | 0.133 | 0.95 | 0.81 | 1.11 | 0.50 | 0.76 | 0.62 | 0.94 | 0.010 |

Appendix 6. Daily mortality rates by temperature groups
* L0: Same-day temperature as visit, L1: temperature one day prior to visit, and so on.

Appendix 7. Results of Autoregressive Distributed Lag (ARDL) models examining the association between extreme temperature (>=31C) and daily mortality rate in emergency department for NCDs.

*Lag 0: Same-day temperature as visit, L1: temperature one day prior to visit, and so on.

|  | HTN | | | | DM | | | | IHD | | | | OLD | | | | CKD | | | | CVA | | | |
| --- | --- | --- | --- | --- | --- | --- | --- | --- | --- | --- | --- | --- | --- | --- | --- | --- | --- | --- | --- | --- | --- | --- | --- | --- |
| Covariates | Coefficient | CI Lower | CI Upper | p-value | Coefficient | CI Lower | CI Upper | p-value | Coefficient | CI Lower | CI Upper | p-value | Coefficient | CI Lower | CI Upper | p-value | Coefficient | CI Lower | CI Upper | p-value | Coefficient | CI Lower | CI Upper | p-value |
| const | 1.51 | 1.43 | 1.58 | <0.001 | 1.86 | 1.77 | 1.96 | <0.001 | 2.27 | 2.15 | 2.39 | <0.001 | 1.63 | 1.46 | 1.80 | <0.001 | 3.39 | 3.08 | 3.71 | <0.001 | 2.74 | 2.42 | 3.07 | <0.001 |
| L0 | 0.06 | -0.27 | 0.40 | 0.71 | 0.23 | -0.19 | 0.65 | 0.29 | 0.74 | 0.19 | 1.30 | 0.01 | 0.08 | -0.69 | 0.85 | 0.85 | 0.07 | -1.31 | 1.45 | 0.92 | 1.30 | -0.12 | 2.72 | 0.07 |
| L1 | 0.49 | 0.10 | 0.89 | 0.01 | 0.20 | -0.30 | 0.69 | 0.43 | -0.15 | -0.80 | 0.49 | 0.64 | 0.29 | -0.60 | 1.19 | 0.52 | 1.04 | -0.54 | 2.62 | 0.20 | -0.30 | -1.91 | 1.30 | 0.71 |
| L2 | 0.01 | -0.38 | 0.40 | 0.96 | 0.19 | -0.30 | 0.68 | 0.44 | 0.25 | -0.39 | 0.89 | 0.45 | 0.17 | -0.73 | 1.06 | 0.71 | 0.53 | -1.05 | 2.11 | 0.51 | -0.13 | -1.74 | 1.48 | 0.88 |
| L3 | 0.32 | -0.07 | 0.71 | 0.11 | 0.40 | -0.09 | 0.90 | 0.11 | -0.04 | -0.68 | 0.61 | 0.91 | 0.32 | -0.58 | 1.22 | 0.48 | 0.44 | -1.15 | 2.02 | 0.59 | -0.40 | -2.01 | 1.21 | 0.63 |
| L4 | 0.09 | -0.30 | 0.48 | 0.65 | 0.20 | -0.29 | 0.69 | 0.43 | -0.01 | -0.66 | 0.63 | 0.97 | -0.26 | -1.16 | 0.64 | 0.57 | 0.05 | -1.53 | 1.64 | 0.95 | 1.38 | -0.23 | 2.99 | 0.09 |
| L5 | 0.35 | -0.04 | 0.74 | 0.08 | -0.04 | -0.53 | 0.45 | 0.88 | 0.47 | -0.17 | 1.12 | 0.15 | -0.20 | -1.09 | 0.70 | 0.66 | -0.27 | -1.85 | 1.31 | 0.74 | 2.20 | 0.59 | 3.81 | 0.01 |
| L6 | -0.34 | -0.73 | 0.05 | 0.09 | -0.26 | -0.75 | 0.23 | 0.30 | -0.33 | -0.98 | 0.31 | 0.31 | 0.35 | -0.54 | 1.25 | 0.44 | 0.02 | -1.56 | 1.61 | 0.98 | 0.16 | -1.45 | 1.77 | 0.84 |
| L7 | 0.31 | -0.02 | 0.65 | 0.07 | 0.42 | 0.00 | 0.85 | 0.05 | 0.56 | 0.00 | 1.11 | 0.05 | 0.57 | -0.20 | 1.34 | 0.15 | 0.03 | -1.35 | 1.41 | 0.97 | -1.12 | -2.55 | 0.30 | 0.12 |

Figure 1. python code

temp = pd.DataFrame(df[['year_adjusted']].value_counts())

for ncd in comorbid_list:

temp = temp.join(pd.DataFrame(df[df[ncd]==True][['year_adjusted']].value_counts()),rsuffix=f'_{ncd}')

temp = temp.div(temp['count'],axis=0)*100

display(temp)

for i,ncd in enumerate(comorbid_list):

sns.lineplot(x='year_adjusted',

y=f'count_{ncd}',

data=temp.sort_index(),

label=ncd,

marker='o',

color=colors_ncd[ncd])

plt.legend(bbox_to_anchor=(1.05, 1), loc='upper left')

plt.ylabel('Visits with NCD per 100 visits')

plt.xlabel('Years')

plt.xticks(rotation=90)

legend=plt.legend()

plt.tight_layout()

plt.savefig('images/fig1.jpg', dpi=600)

plt.show()

Fig 2. Python code

```
import statsmodels.stats.proportion as smp
#visit rate
results = []
for ncd in comorbid_list:
grouped = df.groupby('temp_group').agg(total=(ncd, 'count'), deaths=(ncd, 'sum'))

for temp, row in grouped.iterrows():
n = row['total']
k = row['deaths']
if n > 0:
ci_low, ci_high = smp.proportion_confint(k, n, alpha=0.05, method='wilson')
rate = k / n
results.append({'ncd': ncd, 'temp_group': temp, 'rate': rate*100, 'ci_low': ci_low*100, 'ci_high': ci_high*100})
results_df = round(pd.DataFrame(results), 2)
results_df.to_clipboard(index=False,header=True)
results_df

for i,ncd in enumerate(comorbid_list):
temp = results_df[results_df['ncd']==ncd]
sns.lineplot(x='temp_group',
y=f'rate' ,
marker='o',data=temp.sort_index(),label=ncd, color=colors_ncd[ncd])
plt.fill_between(
x=temp['temp_group'],
y1=temp['ci_low'],
y2=temp['ci_high'],
alpha=0.2,
label=None # Avoid duplicate legend entries
)
print('Visits by temperature')
plt.ylabel('Visits with NCD per 100 Visits')
plt.xlabel('Temperature (°C)')
plt.ylim([0,25])
legend=plt.legend(loc='upper left', bbox_to_anchor=(1.02, 1))
plt.tight_layout()

plt.savefig('images/fig2.jpg', dpi=600)

plt.show()
```

Fig 3. python code

```
import statsmodels.stats.proportion as smp

results = []
for ncd in comorbid_list:
grouped = df[df[ncd]].groupby('temp_group').agg(total=('expired', 'count'), deaths=('expired', 'sum'))

for temp, row in grouped.iterrows():
n = row['total']
k = row['deaths']
if n > 0:
ci_low, ci_high = smp.proportion_confint(k, n, alpha=0.05, method='wilson')
rate = k / n
results.append({'ncd': ncd, 'temp_group': temp, 'rate': rate*100, 'ci_low': ci_low*100, 'ci_high': ci_high*100})
results_df = round(pd.DataFrame(results), 2)
results_df.to_clipboard(index=False,header=True)


for i,ncd in enumerate(comorbid_list):
temp = results_df[results_df['ncd']==ncd]
sns.lineplot(x='temp_group',
y=f'rate' ,
marker='o',data=temp.sort_index(),label=ncd, color=colors_ncd[ncd])
plt.fill_between(
x=temp['temp_group'],
y1=temp['ci_low'],
y2=temp['ci_high'],
alpha=0.1,
label=None # Avoid duplicate legend entries
)
# plt.legend()
# plt.title("Visits by Temperature")
print('Mortality by temperature')
plt.ylabel('Mortality Rate (%)')
plt.xlabel('Temperature (°C)')
plt.ylim([0,6])
legend=plt.legend(loc='upper left', bbox_to_anchor=(1.02, 1))
plt.tight_layout()

plt.savefig('images/fig3.jpg', dpi=600)

plt.show()
```

Appendix 5. python code

```
# Combine all into a single DataFrame
plot_df = pd.concat(plot_data, ignore_index=True)

# display(plot_df)

# Plot
g = sns.FacetGrid(plot_df, col='ncd', height=3, col_wrap=3, sharey=False,sharex=False)
g.map_dataframe(
sns.barplot,
hue='lag',
y='mortality_rate',
x='temp_group',
palette='colorblind'
)

g.set_axis_labels("Temperature (°C)", "Mortality Rate (%)")
g.set_xticklabels(rotation=90)
g.add_legend(title="Lag Days", loc='upper right',bbox_to_anchor=(1.05, 1))
g.set_titles("{col_name}")
# plt.legend(, loc='upper left')

plt.tight_layout()

g.savefig('images/appendix5.jpg', dpi=600)
plt.show()
```

Fig 4. aft python code

```
#visualise
fig, axes = plt.subplots(2, 3, figsize=(10, 6), sharey=True, sharex = True, constrained_layout=True)
axes = axes.flatten() # Flatten to iterate easily

for i, ncd in enumerate(comorbid_list):
temp = results_df[results_df['model'] ==ncd].drop([0,1,6,7,9],axis=0)

temp['Significant'] = temp['p-value'] < 0.05
temp['covariate'] = temp['covariate'].str.replace('temp_group_', '')
# temp = temp.sort_values('covariate')
# # Plot
ax = axes[i]
y_pos = np.arange(len(temp))

ax.hlines(
y=y_pos,
xmin=temp['CI Lower'],
xmax=temp['CI Upper'],
color=colors_ncd[ncd],
linewidth=2
)

# Point estimates
ax.scatter(
temp['Time Ratio'],
y_pos,
s=60,
color=colors_ncd[ncd],
edgecolors=temp['Significant'].map({True: "black", False: colors_ncd[ncd]}),
linewidth=2,
zorder=3
)


# Plot settings
ax.axvline(1, color='black', linewidth=0.8, linestyle='--')
ax.set_ylim(-.5,3.5)
ax.set_xlim(0.6,1.4)
ax.set_yticks(y_pos, labels=temp['covariate'])
# ax.set_yticklabels(temp['covariate'])
ax.set_title(ncd)
# # ax.set_ylim([-1,2])
if i==4:
ax.set_xlabel(f'Time Ratio (± 95% CI)')
if i % 3 == 0:
ax.set_ylabel('Temperature Group (°C)')
if i ==5:
legend_elements = [
Patch(facecolor='lightgrey', edgecolor='black',linewidth=2 ,label='Significant (p < 0.05)'),
Patch(facecolor='lightgrey', edgecolor='black',linewidth = 0.1, label='Not Significant')
]

ax.legend(handles=legend_elements, loc='lower right',bbox_to_anchor=(1, -0.35), borderaxespad=0.)

plt.tight_layout()
fig.savefig('images/fig5.jpg', dpi=600)
plt.show()
```

Figure 5. python code

```
#only >=31
g = sns.FacetGrid(plot_df[plot_df['temp_group']=='>=31'], col='ncd', height=3, col_wrap=3, sharey=False,sharex=False)
g.map_dataframe(
sns.barplot,
x='lag',
y='mortality_rate',
# x='temp_group',
# palette='colorblind'
)

color_map = dict(zip(g.col_names, colors_ncd.values()))

# recolor bars per facet
for ax, ncd in zip(g.axes.flat, g.col_names):
for patch in ax.patches:
patch.set_facecolor(color_map[ncd])

g.set_axis_labels("Lag Days", "Mortality Rate (%)")
# g.add_legend(title="Lag Days", loc='upper right',bbox_to_anchor=(1.05, 1)
g.set_titles("{col_name}")
print('Mortality Rate by Temperature Lag for Extreme Temperatures (>=31°C)')

# plt.legend(bbox_to_anchor=(1.05, 1), loc='upper left')

plt.tight_layout()

g.savefig('images/fig5.jpg', dpi=600)
plt.show()
```

Fig 6. ardl python code

```
#visualise
fig, axes = plt.subplots(2, 3, figsize=(10, 6), sharey=False, constrained_layout=True)
axes = axes.flatten() # Flatten to iterate easily

for i, ncd in enumerate(model_list):
temp = df_ncd_results[ncd]
temp['Significant'] = temp['pvalues'] < 0.05
temp['index'] = temp['index'].str.replace('extreme_temp.', '')
# Plot
ax = axes[i]
edgecolors= temp['Significant'].map({True: "#000000", False: colors_ncd[ncd]})
bars = ax.bar(temp['index'], temp['coef'],
yerr=[temp['coef'] - temp['ci_lower'], temp['ci_upper'] - temp['coef']],
capsize=2,
color=colors_ncd[ncd],
width=0.5,
edgecolor=edgecolors,
linewidth=3)

# Plot settings
ax.axhline(0, color='black', linewidth=0.8, linestyle='--')
ax.set_title(f'{ncd}')
if i==4:
ax.set_xlabel(f'Lag Days of Extreme Temperature ({temp_group}°C)')
if i % 3 == 0:
ax.set_ylabel('Coefficient (± 95% CI)')
if i ==5:
legend_elements = [
Patch(facecolor='lightgrey', edgecolor='black',linewidth=2 ,label='Significant (p < 0.05)'),
Patch(facecolor='lightgrey', edgecolor='black',linewidth = 0.1, label='Not Significant')
]

ax.legend(handles=legend_elements, loc='lower right',bbox_to_anchor=(1, 0.01), borderaxespad=0.)


print('ARDL Coefficients for Extreme Temperature Lag on Mortality Rate by NCD')
# plt.tight_layout()
fig.savefig('images/fig6.jpg', dpi=600)
plt.show()
```
